# Blood product ratio in acute traumatic coagulopathy - effect on mortality in a Scandinavian level 1 trauma centre

**DOI:** 10.1186/1757-7241-18-65

**Published:** 2010-12-07

**Authors:** Jesper Dirks, Henrik Jørgensen, Carsten H Jensen, Sisse R Ostrowski, Pär I Johansson

**Affiliations:** 1 Department of Anesthesia, Centre of Head and Orthopedics, Copenhagen University Hospital, Blegdamsvej 9, DK-2100 Copenhagen, Denmark; 2 Section for Transfusion Medicine, Capital Region Blood Bank, Rigshospitalet, Copenhagen University Hospital, Blegdamsvej 9, DK-2100 Copenhagen, Denmark; 3 Dept. of Anaesthesia and Intensive Care, Hillerød University Hospital, Hillerød, Denmark

## Abstract

**Background:**

Trauma is the leading cause of loss of life expectancy worldwide. In the most seriously injured patients, coagulopathy is often present on admission. Therefore, transfusion strategies to increase the ratio of plasma (FFP) and platelets (PLT) to red blood cells (RBC), simulating whole blood, have been introduced. Several studies report that higher ratios improve survival in massively bleeding patients. Here, the aim was to investigate the potential effect of increased FFP and PLT to RBC on mortality in trauma patients.

**Methods:**

In a retrospective before and after study, all trauma patients primarily admitted to a level-one Trauma Centre, receiving blood transfusion, in 2001-3 (n = 97) and 2005-7 (n = 156), were included. In 2001-3, FFP and PLT were administered in accordance with the American Society of Anesthesiologists (ASA) guidelines whereas in 2005-7, Hemostatic Control Resuscitation (HCR) entailing pre-emptive use of FFP and PLT in transfusion packages during uncontrolled haemorrhage and thereafter guided by thrombelastograph (TEG) analysis was employed. The effect of transfusion therapy and coagulopathy on mortality was investigated.

**Results:**

Patients included in the early and late period had comparable demography, injury severity score (ISS), admission hematology and coagulopathy (27% vs. 34% had APTT above normal). There was a significant change in blood transfusion practice with shorter time interval from admission to first transfusion (median time 3 min vs.28 min in massive bleeders, p < 0.001), transfusion of higher ratios of FFP:RBC, PLT:RBC and PLT:FFP in the HCR group but 30-day mortality remained comparable in the two periods. In the 2005-7 period, higher age, ISS and Activated Partial Thromboplastin Time (APTT) above normal were independent predictors of mortality whereas no association was fund between blood product ratios and mortality.

**Conclusion:**

Aggressive administration of FFP and PLT did not influence mortality in the present trauma population.

## Introduction

Hemorrhage leading to massive transfusion remains a major cause of potentially preventable deaths [1]. Massive transfusion and trauma are associated with the development of coagulopathy, which develops secondary to tissue injury, hypoperfusion, dilution, and consumption of clotting factors and platelets [2]. Coagulopathy, together with hypothermia and acidosis, forms a "lethal triad" associated with a poor prognosis [3]. Furthermore, an acute coagulopathy of trauma and shock (ACoTS) present already at admission the hospital has been identified also being associated with increased mortality [3]. Although the early and effective reversal of coagulopathy is acknowledged to be important, the best method to achieve this goal remains controversial [4].

Recently, the concept of Hemostatic Control Resuscitation (HCR), i.e., providing large transfusions to critically injured patients in an immediate and sustained manner as part of a massive transfusion protocol, has been introduced, with wide implementation of the concept of damage control [3,5]. The rationale behind this hemostatic resuscitation concept is that circulating whole blood contains red blood cells, plasma, and platelets at a 1:1:1 ratio, and transfusion of plasma and platelets in an appropriate unit-for-unit ratio has been proposed as a way to both prevent and treat coagulopathy due to massive hemorrhage. A number of retrospective studies have reported the benefit on survival in trauma patients receiving high ratios of fresh frozen plasma (FFP) and platelet concentrates (PLT) in relation to red blood cells (RBC) when compared to those receiving less FFP and PC [6].

At Rigshopitalet, Haemostatic Control Resuscitation (HCR) encompassing preemptive use of PLT and FFP in tailored transfusion packages immediately upon arrival at the trauma centre with subsequent transfusion therapy directed by the results of thrombelastograph (TEG) analysis throughout the peri- and postoperative period was implemented in 2004 [7] and the aim of the present study was to investigate the potential effect of HCR on mortality in trauma patients when compared to those treated before the implementation of HCR.

## Methods

We undertook a before and after study using historical controls. Patients treated in 2001-3 were compared to patients treated in 2005-7. 2004 was excluded, since HCR for massively bleeding patients was introduced this year, as previously described [7]. In brief, HCR was introduced including the following services: (i) transfusion packages comprising 5 units of RBCs stored in saline-adenine-glucose-manitol (SAGM) for a maximum of 15 days, 5 units of FFP and 2 units of PLT (buffy coat pool from four donors), to be used before the results of the TEG analysis was available; (ii) storage of thawed, ready-to-use FFP in the blood bank for a maximum of 72 h; (iii) continuous monitoring of the blood transfusion therapy in patients receiving more than 10 RBCs within 24 h; (iv) protocol for monitoring of haemostatic competence with TEG and an intervention algorithm for treatment with FFP and PLT based on the results of the analysis (Appendix 1); and (v) educational program for anesthesiologists concerning functional hemostasis and TEG.

All Consecutive trauma patients admitted to the Trauma Centre, Copenhagen, Rigshospitalet in 2001-3 (n = 1448) and 2005-7 (n = 2553) were identified. All secondary transfers were excluded. All patients receiving ≥1 blood product at admission were then identified by merging data from all trauma patients admitted to the Trauma Centre with data from the blood bank of all patients receiving blood 2001-3 (n = 120) and 2005-7 (n = 209). ISS scores were obtained from the Trauma Audit & Research Network (TARN) data base, and only patients with available ISS were included, which reduced the number of patients to 97 (2001-3) and 156 (2005-7). Admission blood samples were collected from the laboratory data base. LOS and 30 day mortality were obtained from the database of the hospital and the Central Office of Civil Registration. All data were collected and entered into a study database based on unique personal identity number after approval from the Data Protection agency. The resulting database contained ISS, age, gender, time from arrival to first blood product delivery, type and amount of blood products (RBC, FFP and PLT) in the first 6 hours, 6-12, 12-24 hours and total amount during hospital stay, admission hematology and coagulation, LOS and mortality. In the present study, coagulopathy was defined as APTT (or INR) just above normal reference value, which is in accordance with the increase in mortality recently reported by Frith et al [8] though the authors here used a, for the study created, prothrombin time ratio. Given the increase in mortality with standard coagulation tests just above normal [8] and the previously reported stronger prognostic value of PTT as compared to PT in trauma patients [9] we chose to define coagulopathy as APTT above normal reference value.

The regional ethics committee of Copenhagen approved the waiver of consent, as all procedures were part of standard care.

## Statistics

Data on patients stratified according to study period or mortality were compared by Wilcoxon Rank Sum and Chi-square test. Early factors associated with blood transfusion within each period were investigated by Spearman correlations, presented by rho and p-values, and differences in these factors between periods were investigated by analysis of covariance (ANCOVA) by including an interaction between period*variable in each model. Furthermore, we investigated factors associated with massive transfusion (MT) by logistic regression analysis, with MT (RBC >10 day 1, n = 66) as dependent variable.

Survival analysis was performed with death as the main endpoint. Follow-up times were calculated from admission date to date of death or censored as alive by the 1 June 2010. Since ~90% of trauma deaths occurred within the first 30 days, only 30-day mortality is reported here. Thirty-day mortality in risk-stratified patients was performed by the Kaplan-Meier method and log-rank test, presented with Chi-square and p-values. Cox proportional-hazards models were done to determine the predictive value for mortality of ISS, age, admission hematology and coagulopathy and early blood transfusion therapy. Significant univariate variables were included in subsequent multivariate models, presented by hazards ratios (HR) with 95% confidence intervals (CI) and p-values. Cases in the two periods were not matched.

Data are presented as medians with inter quartile ranges (IQR) unless otherwise stated. P-values < 0.05 were considered significant. Statistical calculations were performed using SAS 9.1 (SAS Institute Inc., Cary, NC, US) and Kaplan-Meier plots performed using WinSTAT^® ^for Microsoft^® ^Excel version 2009.1 (R. Fitch Software).

## Results

### Study patients

A total of 120 and 209 patients from the early (2001-3) and late (2005-7) period, respectively, were identified according to the admission and blood transfusion criteria, but 22 (early period) and 51 (late period) of these were excluded due to missing ISS, leaving a total of 253 patients in the study: 97 from the 2001-3 period and 156 from the 2005-7 period (Table 1). The excluded patients from each of two periods were comparable with regards to age (p = 0.468), gender (p = 0.429) and mortality (p = 0.491). When comparing the excluded patients to those included (n = 253), the two groups had comparable age, gender distribution, early and late blood transfusion requirements, blood product ratios, hemoglobin, platelet count, APTT and INR. The only variable that differed between the two groups was time to first blood product transfusion (median 44 min in patient included vs. 16 min in patients excluded) probably reflecting that more patients from the 2005-7 period were excluded.

**Table 1:** Demography and admission hematology and coagulopathy in the 253 trauma patients included in the study from the 2001-3 and 2005-7 periods.

		Study period 2001-3	Study period 2005-7	**P-value**^**1**^
n		97	156	
Age	yrs	40 (28-55)	43 (27-59)	0.622
Gender	male	66 (74%)	116 (72%)	0.254
				
ISS	score	20 (13-30)	24 (16-34)	0.289
	group 0 (0-15)	31 (32%)	32 (20%)	0.123
	group 1 (16-27)	36 (37%)	68 (44%)	
	group 2 (28-75)	30 (31%)	56 (36%)	
				
Mortality^2^	deceased	24 (25%)	47 (31%)	0.382
				
Hospital LOS	all (days)	18 (8-41)	18 (3-35)	0.334
	survivors (days)	23 (11-54)	26 (15-51)	0.618
	deceased (days)	2 (2-15)	2 (1-3)	**0.019**
				
Hemoglobin	mmol/l	6.7 (5.6-7.4)	6.9 (5.8-7.7)	0.059
Leukocyte count	*10^9^/l	12 (9-15)	12 (8-16)	0.707
				
Platelet count	*10^9^/l	190 (130-240)	186 (123-249)	0.775
	< 150*10^9^/l	34%	31%	0.650
APTT	s	31 (28-37)	31 (27-39)	0.980
	> 35 s	27%	34%	0.273
INR	ratio	1.2 (1.1-1.4)	1.2 (1.1-1.3)	0.256
	> 1.2	40%	44%	0.183

^1^Patients from the 2001-3 period and 2005-7 period were compared by Wilcoxon Rank Sum and Chi-square test. ^2^Total mortality related to the trauma. Data are presented as medians (IQR), n (%) or %, with p-values in bold for variables with p < 0.05.

The patients included from period 2001-3 and 2005-7 displayed comparable demography, injury severity, admission hematology and coagulopathy (Table 1). Hospital LOS was shorter in deceased patients in 2005-7, and there was a trend towards lower hemoglobin level in 2001-3 and higher ISS in 2005-7. A total of 27-34% of the patients had APTT above normal and hence coagulopathy on admission. The vast majority of patients sustained blunt trauma (approximately 85% reported in previous studies of patients admitted to the Trauma Centre at Rigshospitalet [10]) and the proportion of penetrating trauma were comparable in the two periods (personal communication, senior thoracical surgeon).

### Blood transfusion therapy

Blood transfusion therapy changed significantly from the early to the late study period, with shorter time interval from admission to first transfusion, transfusion of more FFP and PLT with higher ratios of FFP:RBC, PLT:RBC and PLT:FFP early (0-6 h) and in total (Table 2). The subgroups of massively bleeding patients (MT, >10 RBC the initial 0-24 h) from 2001-3 and 2005-7 had comparable demography, ISS, admission hematology and coagulopathy and mortality though patients in 2005-7 received transfusions faster and with more RBC, FFP, PLT in higher ratios (Table 3). The proportion of MT patients in the early (22%) and late (29%) period was comparable (p = 0.279). In the univariate analysis, variables associated with MT were higher ISS (p < 0.001), decreased time to first blood product (p < 0.001), lower hemoglobin (p = 0.014), lower platelet count (p = 0.026) and higher APTT (p = 0.001) and increased amounts of FFP 0-6 h (p < 0.001), PLT 0-6 h (p < 0.001), increased FFP/RBC ratio 0-6 h (p = 0.001), increased PLT/RBC ratio 0-6 h (p = 0.004), whereas period (p = 0.280), age (p = 0.860), FFP/PLT-ratio 0-6 h (p = 0.381) and INR (p = 0.721) were not associated with MT. In a multivariate model including ISS and time to first blood product, lower hemoglobin (p = 0.032), lower platelet count (p = 0.041) and higher APTT (p = 0.017) and increased amounts of FFP 0-6 h (p < 0.001) and PLT 0-6 h (p < 0.001) were independently associated with MT whereas ratios were not independently associated with MT (data not shown).

**Table 2:** Blood transfusion therapy in each study period (2001-3 and 2005-7).

		Study period 2001-3	Study period 2005-7	**P-value**^**1**^
Time to first blood product	min	85 (33-151)	26 (2-72)	**< 0.001**
				
RBC 0-6 h	n	4 (2-10)	5 (2-12)	0.280
RBC total	n	5 (2-10)	5 (3-12)	0.355
FFP 0-6 h	n	0 (0-4)	3 (0-10)	**< 0.001**
FFP total	n	0 (0-4)	3 (0-10)	**< 0.001**
PLT 0-6 h	n	0 (0-0)	1 (0-4)	**< 0.001**
PLT total	n	0 (0-0)	1 (0-4)	**< 0.001**
				
FFP:RBC 0-6 h	ratio^2^	0 (0-0.33)	0.56 (0-0.83)	**< 0.001**
FFP:RBC total	ratio^2^	0 (0-0.36)	0.60 (0-0.83)	**< 0.001**
PLT:RBC 0-6 h	ratio^2^	0 (0-0)	0.17 (0-0.33)	**< 0.001**
PLT:RBC total	ratio^2^	0 (0-0)	0.17 (0-0.33)	**< 0.001**
PLT:FFP 0-6 h	ratio^2^	0 (0-0.09)	0.40 (0.21-0.43)	**< 0.001**
PLT:FFP total	ratio^2^	0 (0-0.14)	0.40 (0.2-0.47)	**< 0.001**

^1^Patients from '2001-3 and 2005-7 were compared by Wilcoxon Rank Sum test. Data are presented as medians (IQR), with p-values in bold for variables with p < 0.05. ^2^A ratio of 0.56, 0.60, 0.17 and 0.40 corresponds to 1.8, 1.7, 5.9 and 2.5, respectively, RBC per FFP or PLT.

**Table 3:** Demography, outcome, admission hematology, coagulopathy and blood transfusion in massively transfused patients (> 10 RBC the initial 0-24 h) in each study period (2001-3 and 2005-7).

		Study period 2001-3	Study period 2005-7	**P-value**^**1**^
n		21	45	
Age	years	50 (35-69)	40 (28-52)	0.106
Gender	male	16 (76%)	32 (71%)	0.666
				
ISS	score	26 (17-34)	27 (19-36)	0.694
				
Mortality	deceased	7 (37%)	17 (39%)	0.893
				
Hemoglobin	mmol/l	5.7 (4.6-6.8)	6.7 (5.6-7.6)	0.066
Platelet count	< 150*10^9^/l	8 (53%)	17 (44%)	0.520
APTT	> 35 s	9 (60%)	24 (62%)	0.917
INR	> 1.2	11 (73%)	21 (54%)	0.192
				
Time to first blood product	min	28 (22-65)	3 (0-23)	**< 0.001**
				
RBC 0-6 h	n	12 (12-24)	20 (14-30)	**0.0394**
RBC total	n	14 (12-23)	20 (14-30)	0.0933
FFP 0-6 h	n	5 (4-10)	14 (10-20)	**< 0.001**
FFP total	n	6 (4-12)	14 (10-21)	**< 0.001**
PLT 0-6 h	n	0 (0-1)	6 (4-8)	**< 0.001**
PLT total	n	0 (0-2)	6 (4-8)	**< 0.001**
				
FFP:RBC 0-6 h	ratio	0.33 (0.16-0.53)	0.71 (0.59-0.85)	**< 0.001**
FFP:RBC total	ratio	0.36 (0.24-0.59)	0.74 (0.61-0.85)	**< 0.001**
PLT:RBC 0-6 h	ratio	0 (0-0.05)	0.29 (0.20-0.37)	**< 0.001**
PLT:RBC total	ratio	0 (0-0.05)	0.29 (0.20-0.35)	**< 0.001**
PLT:FFP 0-6 h	ratio	0 (0-0.10)	0.40 (0.36-0.43)	**< 0.001**
PLT:FFP total	ratio	0 (0-0.13)	0.40 (0.35-0.46)	**< 0.001**

^1^Patients from period 1 and 2 were compared by Wilcoxon Rank Sum test. Data are presented as medians (IQR), with p-values in bold for variables with p < 0.05.

### Early factors associated with blood transfusion

#### Time to first blood transfusion

**Figure 1:**
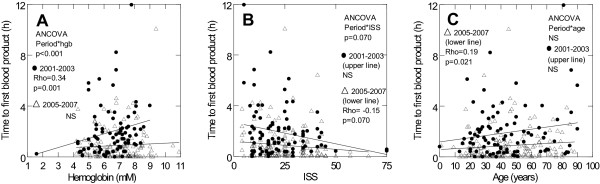
**Early Transfusion Triggers in Trauma Patients**. Scatter plots displaying the correlation between potential early factors contributing to the decision to transfuse blood products in patients admitted to the trauma centre, rigshospitalet, copenhagen university hospital, denmark, in the period 2001-3 and 2005-7. For each period the plots show correlations between: a) hemoglobin (mm) and time to first blood product delivery (h), b) age (years) and time to first blood product delivery (h) and c) iss and time to first blood product delivery (h). The y-axis is truncated at 12 hours leaving out 4 observations that though not displayed contribute to the statistics. Rho and p-values are shown for each period together with p-values for the ancova (period*variable interaction).

In 2001-3, the time to first blood transfusion correlated positively with hemoglobin (Figure 1A) whereas it tended to correlate negatively with ISS (Figure 1B) and correlated positively with age (Figure 1C) in 2005-7, with a significant period-interaction with regards to hemoglobin (p < 0.001) and a trend with regards to ISS (p = 0.070). In massive bleeders, however, the time to first transfusion did not correlate with hemoglobin, ISS or age in any of the periods (data not shown).

#### Blood transfusions 0-6 h

**Figure 2:**
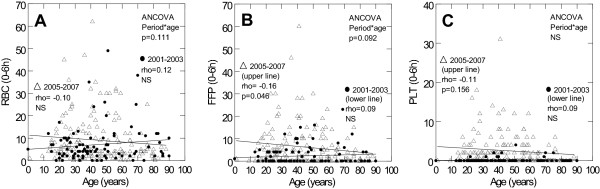
**Age and Blood Product Use in Trauma Patients**. scatter plots displaying potential early blood transfusion triggers in patients admitted to the trauma centre, rigshospitalet, copenhagen university hospital, denmark, in the period 2001-3 and 2005-7. for each period the plots show correlations between: a) age (years) and rbc 0-6 h (n), b) age (years) and ffp 0-6 h (n) and c) age (years) and plt 0-6 h (n). rho and p-values are shown for each period together with p-values for the ancova (period*variable interaction).

In 2005-7, the number and ratios of blood transfusions 0-6 h correlated positively with ISS for FFP (rho = 0.25, p = 0.002), PLT (rho = 0.30, p < 0.001), FFP:RBC (rho = 0.26, p = 0.001), FFP:RBC (rho = 0.30, p < 0.001) and PLT:FFP (rho = 0.17, p = 0.093), but negatively with age for FFP (Figure 2B) and FFP:RBC (rho = -0.25, p = 0.002). In 2001-3, neither ISS nor age correlated with numbers or ratios of blood transfusions 0-6 h (Figure 2A-C, data not shown for ISS and ratios).

Significant period-interactions between ISS and blood transfusions 0-6 h were found for FFP (p = 0.002), PLT (p = 0.002), FFP:RBC (p = 0.033) and PLT:RBC (p = 0.024). There was a trend towards period-interactions between age and blood transfusions 0-6 h for RBC and FFP (Figure 2A-B). In massive bleeders, age did not correlate with blood transfusion therapy in any period whereas ISS correlated positively with RBC, FFP, PLT, FFP:RBC and PLT:RBC 0-6 h in 2005-7 (data not shown).

#### Coagulopathy

In both periods, platelet count correlated negatively with FFP, PLT and ratios of FFP:RBC, PLT:RBC and PLT:FFP 0-6 h (data not shown). APTT correlated negatively with time to first blood transfusion and positively with number of transfused RBC, FFP and PLT 0-6 h (both periods) and FFP:RBC, PLT:RBC and PLT:FFP ratios (only 2005-7) (data not shown).

### Mortality

**Figure 3:**
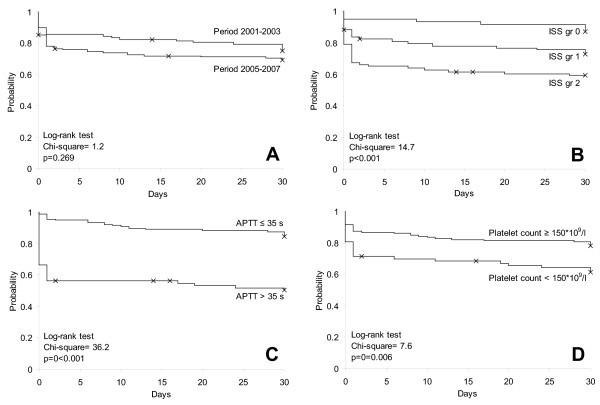
**Survival in Trauma Patients Stratified According to Period, Iss and Coagulopathy Measures**. kaplan-meier plots showing 30-day mortality in trauma patients admitted to the trauma centre, rigshospitalet, copenhagen university hospital, denmark, in the period 2001-3 and 2005-7 stratified according to: a) period (2001-3 vs. 2005-7), b) iss group (0 (iss 0-15), 1 (iss 16-27) and 2 (28-75)), c) aptt (> 35 s vs. ≤35 s) and d) platelet count (< 150 *10^9^/l vs. ≥150 *10^9^/l). survival was compared by log-rank test, with p-values and chi-squares shown.

The overall 30-day mortality did not differ between study periods (Figure 3A, Table 1). In both periods, deceased patients had higher ISS (27 (120-38) vs. 20 (13-29), p < 0.001, Figure 3B), higher APTT (39 (30-66) vs. 30 (27-35), p < 0.001, Figure 3C), lower platelet count (166 (101-212) vs. 198 (141-255), p = 0.003, Figure 3D) and higher age (55 (43-70) vs. 37 (25-50), p < 0.001) compared to survivors. The same differences were observed between massively transfused survivors and deceased patients (data not shown).

Overall mortality within each ISS stratum was 13%, 28% and 42%, respectively (mortality in ISS group 0, 1 and 2 in the 2001-3 period was 19%, 24% and 34% whereas it in the 2005-7 period was 6%, 30% and 45%). Furthermore, overall mortality was 49% and 16% in patients with or without coagulopathy according to APPT and it was 38% and 22% in thrombocytopenic vs. non-thrombocytopenic patients (Figure 3B-D). The only difference in transfusion therapy between survivors and deceased patients was a higher number of transfused RBC 0-6 h in deceased patients (5 (3-17) vs. 4 (2-9), p = 0.044).

#### Cox Proportional-hazards models

In Cox analyses including all patients, higher ISS, age, transfused RBC and PLT 0-6 h, platelet count and APTT above normal were all associated with higher mortality (Table 4 upper part) but only ISS, age and APTT were independent predictors of mortality (Table 4).

**Table 4:** Univariate and multivariate Cox proportional-hazards models for a composite of both periods and within each period (2001-3, 2005-7).

		Univariate		Multivariate	
		**HR (95% CI)**^**1**^	P-value	**HR (95% CI)**^**1**^	P-value
**Study period 2001-3 and 2005-7 (n = 220)**^**7**^					
ISS	score^2^	1.03 (1.01-1.06)	**0.001**	1.03 (1.01-1.06)	**0.007**
Age	years^3^	1.04 (1.02-1.05)	**< 0.001**	1.04 (1.03-1.06)	**< 0.001**
RBC 0-6 h	n^4^	1.03 (1.01-1.05)	**0.013**	1.01 (0.96-1.05)	0.756
FFP 0-6 h	n^4^	1.02 (1.00-1.05)	0.055	0.94 (0.84-1.06)	0.329
PLT 0-6 h	n^4^	1.06 (1.01-1.11)	**0.014**	1.12 (0.92-1.36)	0.246
Platelet count	10*10^9^/l^5^	0.96 (0.93-0.99)	**0.007**	0.99 (0.96-1.02)	0.353
APTT	> 35 s^6^	4.60 (2.69-7.88)	**< 0.001**	3.09 (1.61-5.93)	**< 0.001**
**Study period 2001-3 (n = 79)**^**7**^					
ISS	score^2^	1.01 (0.97-1.05)	0.646	1.02 (0.98-1.07)	0.350
Age	years^3^	1.05 (1.02-1.07)	**< 0.001**	1.05 (1.03-1.08)	**< 0.001**
RBC 0-6 h	n^4^	1.01 (0.95-1.07)	0.817	0.97 (0.88-1.07)	0.520
FFP 0-6 h	n^4^	0.94 (0.80-1.11)	0.474	0.70 (0.49-0.99)	**0.045**
PLT 0-6 h	n^4^	1.20 (0.63-2.26)	0.582	5.30 (0.79-35.32)	0.085
Platelet count	10*10^9^/l^5^	0.97 (0.92-1.03)	0.371	0.97 (0.91-1.02)	0.247
APTT	> 35 s^6^	3.42 (1.28-9.15)	**0.014**	2.47 (0.66-9.21)	0.177
**Study period 2005-7 (n = 141)**^**7**^					
ISS	score^2^	1.04 (1.02-1.07)	**0.001**	1.04 (1.01-1.07)	**0.020**
Age	years^3^	1.03 (1.01-1.05)	**< 0.001**	1.04 (1.02-1.06)	**< 0.001**
RBC 0-6 h	n^4^	1.03 (1.01-1.05)	**0.016**	1.04 (0.97-1.12)	0.223
FFP 0-6 h	n^4^	1.02 (1.00-1.05)	0.085	0.98 (0.87-1.11)	0.749
PLT 0-6 h	n^4^	1.05 (1.00-1.10)	0.061	0.95 (0.76-1.19)	0.676
Platelet count	10*10^9^/l^5^	0.95 (0.92-0.99)	**0.011**	0.98 (0.95-1.02)	0.320
APTT	> 35 s^6^	5.04 (2.62-9.68)	**< 0.001**	3.41 (1.55-7.51)	**0.002**

^1^Relative hazards with 95% confidence intervals (HR (95% CI)) and p-values are shown for all variables, with p-values in bold for variables with p < 0.05. ^2^One unit increase in ISS, ^3^One years older, ^4^One more unit of the respective blood component, ^5^A 10*10^9^/l increase in platelet count, ^6^APTT > 0.35 s (above reference range). ^7^Each univariate and multivariate Cox models only included patients with complete data (i.e., the same patients were included in each univariate and multivariate analyses)

In 2001-3, higher age and lower numbers of FFP 0-6 h were the only independent predictors of higher mortality whereas ISS, age and APTT were independent predictors of mortality in 2005-7 (Table 4 middle and lower part). When only including univariately significant variables in the multivariate Cox analysis (and not forcing ISS, product use and platelet count into the model) in the 2001-3 period, higher age and APTT were the only independent predictors of mortality here.

In massively transfused patients (n = 66), higher ISS (p = 0.073), age (p = 0.098) and APTT above normal (p = 0.011) were (borderline) significant univariate predictors of mortality but only age (HR 1.03 (1.0-1.1), p = 0.043) and APTT above normal (HR 4.9 (1.1-22.6), p = 0.040) independently predicted mortality whereas ISS did not (data not shown). In patients with APTT above normal (n = 71), higher age and APTT, but not ISS, were independent predictors of mortality whereas in patients with normal APTT (n = 155), only higher age and ISS, but not APTT, independently predicted mortality (data not shown).

When the multivariate models (including age, ISS and APTT) for massive bleeders and patients stratified according to APTT were confronted with RBC, FFP, PLT, FFP:RBC, PLT:RBC and PLT:FFP 0-6 h, this did not change the results (data not shown).

## Discussion

The main finding of the present study was that a change in transfusion therapy with more aggressive and early administration of plasma and platelets in relation to RBC did not influence survival in the trauma patients investigated, which was also confirmed by multivariate analysis in massively transfused patients.

Recently a substantial number of retrospective studies assessing the influence of ratios of FFP and PLT in relation to RBC have been published in trauma patients reporting on the benefit of ratios approximating 1:1:1 [11-14], which contrasts the findings in the present study. Potential explanation for this difference may be related to the fact that the present study was a before-and- after study where a substantial change in transfusion therapy was implemented, whereas retrospective evaluations not introducing a shift in transfusion practice have previously been reported. Also, a substantial number of studies report on findings from the combat scene and thereby a different kind of trauma patients with higher frequency of penetrating injuries than present in the current study. Our findings however concur with Scalea et al., reporting no survival benefit in patients receiving high FFP:RBC and PLT:RBC-ratios at a major civilian trauma center [15]. In contrast to the present study, Cotton et al. reported a reduction in mortality after introduction of a massive transfusion (MT) protocol in group of MT patients [16] and in another study reported a reduction in multiple organ failure (MOF) and postinjury complications in patients transfused according to the MT protocol, though no change in mortality was reported [17]. Given that conclusions based on retrospective studies like the present are associated with survival (and mortality) bias as compared to conclusions based on prospective efficacy studies, the results presented here should be interpreted with caution.

It has previously been reported that not only the ratio of FFP:RBC and PLT:RBC are important for survival but also the timing of the administration of FFP and PLT, as patients receiving early FFP and PLT therapy displayed improved survival [18]. In the present study, administration of FFP and PLT commenced within the first five min after arrival at the trauma center in the late period as compared to 28 min in the early period, but this did clearly not improve survival in this cohort of patients. It should however be noted, that in the study by Riskin et al. patients receiving early administration of blood transfusions transfusion commenced much later than those receiving transfusions late in the present study. The lack of improvement of survival in trauma patients in the present study contrasts the finding in patients undergoing surgery for a ruptured abdominal aortic aneurysm (rAAA) previously reported [19], which may be related to the different extent of tissue injury between these cohorts. In the present study approximately 30% of the patients demonstrated coagulopathy at admission as evaluated by APTT>35 s, which was associated with a 3-fold increase in mortality in accordance with that previously reported by Brohi et al.[20,21]. Patients with a rAAA rarely present with coagulopathy upon admission [19] thus supporting the notion that the bleeding pathophysiology of these patients differ from that in severely injured trauma patients. In the present study, APTT was a strong and independent predictor of higher mortality in massively transfused patients, and even higher APTT in patients presenting with coagulopathy (APTT above normal) was an independent predictor of mortality whereas APTT could not predict mortality in patients presenting with a normal APTT. The findings of the present study could indicate that the devastating effects of trauma and subsequent hypoperfusion occurring immediately after the trauma and before arrival at the trauma center may not be reversed by transfusion therapy alone despite achievement of normal haemostatic competence early in the resuscitation phase, as previously reported [22].

Furthermore, earlier transfusion and increased amounts of FFP and PLT did in this study not reduce the rate of MT patients since this was comparable in the two periods. However, due to the retrospective nature of this study, a cause-effect relationship between MT and different variables cannot be established.

Interestingly, we found that in the early period hemoglobin was the main factor that triggered early blood transfusion whereas higher ISS (or injury severity since ISS is a derived figure that was not available at the time point of admission) was a significant factor that triggered early transfusion in the late period. Furthermore, higher age was in the late period associated with longer time to first transfusion and transfusion of less FFP and hence a lower FFP:RBC ratio, indicating that patients with an advanced age received less aggressive transfusion therapy, which not is recommended in the hospital transfusion guidelines and consequently an effect introduced by the treating physicians. It is, however, unclear whether this practice negatively affected outcome in these individuals since in all groups studied, age was independently associated with outcome which is in alignment with previous reports [9,23]. The negative predictive value of higher age for survival following trauma is likely explained in part by the increase in co-morbidity and a higher frequency of patients on medications with advanced age, which may negatively influence hemostasis [24] and cardiovascular adaptability. Furthermore, it is well established that systemic inflammatory response syndrome (SIRS) is a particularly serious problem in the aging population and this relates to increased production of pro-inflammatory cytokines [25,26]. Recently, it was reported that advanced age is associated with a decrease in thrombomodulin and activated protein C in an animal model, suggesting that also the anticoagulation system is negatively affected by older age, making the individual more pro-thrombotic [27]. The findings of the present study however demonstrate that the negative predictive value of advanced age for survival is independent of presence or absence of coagulopathy, indicating that more general impairment of adaptation mechanisms may explain the excess mortality during critical illness including ACoTS.

This study has several limitations. Obviously it is a retrospective study not a prospective, the injury pattern (blunt vs. penetrating) has not been investigated, the amount of infused prehospital fluid has not been stated and might differ due to restrictive fluid resuscitation in period two. Also, given the limited number of patients included in this study the exclusion of more than twice the number of patients in the late group as compared to the early group may have influenced the results presented considerably.

In conclusion, the present study demonstrated that a change in transfusion therapy with more aggressive and early administration of FFP and PLT in relation to RBC did not influence survival in the trauma patients investigated, indicating that the devastating effects of trauma and subsequent hypoperfusion cannot be reversed by transfusion therapy alone. Prospective studies addressing the effect of various means of Hemostatic Control Resucitation in trauma patients presenting bleeding requiring transfusion are desperately needed.

## Competing interests

The authors declare that they have no competing interests.

## Authors' contributions

JD, HJ and CHJ: have made substantial contributions to conception and design, acquisition of data, analysis and interpretation of data; SRO: has made substantial contributions to analysis and interpretation of data; PIJ has made substantial contributions to conception and design, acquisition of data, analysis and interpretation of data. All authors have been involved in drafting the manuscript and have given final approval of the version to be published.
